# Social Cognitive Role of Schizophrenia Candidate Gene *GABRB2*

**DOI:** 10.1371/journal.pone.0062322

**Published:** 2013-04-24

**Authors:** Shui Ying Tsang, Songfa Zhong, Lingling Mei, Jianhuan Chen, Siu-Kin Ng, Frank W. Pun, Cunyou Zhao, Bingyi Jing, Robin Chark, Jianhua Guo, Yunlong Tan, Lijun Li, Chuanyue Wang, Soo Hong Chew, Hong Xue

**Affiliations:** 1 Division of Life Science and Applied Genomics Laboratory, Hong Kong University of Science and Technology, Clear Water Bay, Hong Kong, China; 2 Department of Economics, Hong Kong University of Science and Technology, Clear Water Bay, Hong Kong, China; 3 Department of Economics, National University of Singapore, Singapore, Rep. of Singapore; 4 Center for Statistical Science, Hong Kong University of Science and Technology, Clear Water Bay, Hong Kong, China; 5 Department of Mathematics, Hong Kong University of Science and Technology, Clear Water Bay, Hong Kong, China; 6 Department of Marketing, National University of Singapore, Singapore, Rep. of Singapore; 7 School of Mathematics and Statistics, Northeast Normal University, Changchun, China; 8 Beijing Huilongguan Hospital, Beijing, China; 9 Beijing Anding Hospital, Capital Medical University, Beijing, China; 10 State Key Laboratory of Molecular Neuroscience, Hong Kong University of Science and Technology, Clear Water Bay, Hong Kong, China; University of Illinois at Chicago, United States of America

## Abstract

The occurrence of positive selection in schizophrenia-associated *GABRB2* suggests a broader impact of the gene product on population fitness. The present study considered the possibility of cognition-related *GABRB2* involvement by examining the association of *GABRB2* with psychosis and altruism, respectively representing psychiatric and psychological facets of social cognition. Four single nucleotide polymorphisms (SNPs) were genotyped for quantitative trait analyses and population-based association studies. Psychosis was measured by either the Positive and Negative Syndrome Scale (PANSS) or antipsychotics dosage, and altruism was based on a self-report altruism scale. The minor alleles of SNPs rs6556547, rs1816071 and rs187269 in *GABRB2* were correlated with high PANSS score for positive symptoms in a Han Chinese schizophrenic cohort, whereas those of rs1816071 and rs1816072 were associated with high antipsychotics dosage in a US Caucasian schizophrenic cohort. Moreover, strongly significant *GABRB2*-disease associations were found among schizophrenics with severe psychosis based on high PANSS positive score, but no significant association was observed for schizophrenics with only mild psychosis. Interestingly, in addition to association with psychosis in schizophrenics, rs187269 was also associated with altruism in healthy Han Chinese. Furthermore, parallel to correlation with severe psychosis, its minor allele was correlated with high altruism scores. These findings revealed that *GABRB2* is associated with psychosis, the core symptom and an endophenotype of schizophrenia. Importantly, the association was found across the breadth of the psychiatric (psychosis) to psychological (altruism) spectrum of social cognition suggesting *GABRB2* involvement in human cognition.

## Introduction

Schizophrenia (SCZ) is a common, multi-factor psychiatric disorder characterized by a strong genetic component, with heritability of around 0.81 [1]. SCZ is clinically heterogeneous and this may reflect underlying genetic heterogeneity. Multiple susceptibility genes for SCZ include *DISC* [2], *COMT* [3], *NRG1* [4] and *GABRB2* encoding the GABA_A_ receptor β_2_ subunit gene for which we reported SCZ associations for single nucleotide polymorphisms (SNPs) and haplotypes in Introns 8 and 9 in a Chinese population-based study [5]. This SCZ-*GABRB2* association was subsequently validated by us using German and Japanese sample cohorts [6], [7] as well as by other researchers using additional samples from Chinese, German and Portuguese cohorts [8], [9]. Contribution of *GABRB2* to SCZ etiology was further supported by the differential expression of different splicing variants of the gene in SCZ patients relative to control subjects [10], [11]. Recently, evidence for imprinting of *GABRB2* was revealed [12], supporting the involvement of genomic imprinting in the development of SCZ [13]. That the disorder persists in the face of its reproductive disadvantage poses an apparent evolutionary paradox [14], and the findings of co-occurrence of recombination and positive selection in this region of *GABRB2* [15], [16] has shed some insight into potential genetic mechanisms underlying the phenomenon. Moreover, the positive selection in *GABRB2* suggests that the GABA_A_ receptor β_2_ subunit may exert a broader impact beyond SCZ morbidity.

Psychosis (referring to psychotic symptoms and not to psychotic disorders) features prominently in many neuropsychiatric diseases including SCZ, bipolar disorder (BPD) and neurodegenerative disorders like Alzheimer’s disease. It has been associated also with impaired cognitive function [17] as well as social cognition [18], [19]. In biological terms, over-activity of the mesolimbic dopamine pathway has been suggested as a contributor to the positive symptoms of psychosis including delusions and hallucinations; a number of genes in the dopamine pathway including *COMT* [20] and *DTNBP1* [21] showed association with risk of psychosis. Other SCZ-associated genes were reported to be involved in psychotic susceptibility in different psychiatric disorders [22], [23], [24], [25], which included *GABRB2* association with psychotic risk in BPD [26], suggesting that genetic modulation operates more on the psychosis endophenotype level rather than being localized to any particular psychiatric diagnosis. In keeping with this, a number of candidate susceptibility genes for psychiatric disorders were found to be involved in normal cognition-related behavior [27], [28], [29], [30], [31], suggesting that these genes could regulate both typical and impaired cognitive processes. Indeed, for prosocial behavior such as empathy, cooperativeness and altruism, where social cognition constitutes an essential component, heritability estimates of about 50% were obtained [32], with possible involvement of the dopaminergic system [33]. Since intricate interactions occur between the dopaminergic and GABAergic systems, genes in the GABA pathway could well participate in such cognition-related phenotypes as psychosis and prosocial behavior.

In the present study, the possible correlation between psychosis and SNPs in the SCZ-associated *GABRB2* gene was examined with respect to the severity of psychosis assessed on the basis of Positive and Negative Syndrome Scale (PANSS) [34] and antipsychotics treatment dosages administered to SCZ patients. In parallel, the possible association of these SNPs with prosocial altruistic behavior was evaluated in healthy subjects to explore any genetic overlap between psychosis and altruism, respectively representing psychiatric and psychological facets of social cognition, regarding their relationships with *GABRB2*.

## Materials and Methods

### Ethics Statement

Written consent was obtained from all subjects prior to the study. Approval for the study was obtained from the ethical committees of Beijing Anding Hospital, Beijing Huilongguan Hospital and Beijing Normal University.

### DNA Samples

For the disease-association studies, Han Chinese patients were recruited from the psychiatric wards of Beijing Anding Hospital and Beijing Huilongguan Hospital, and control subjects were region-matched Han Chinese. The patients were diagnosed according to the Diagnostic and Statistical Manual of Mental Disorders (DSM)-IV criteria [35] and additionally assessed for PANSS scores. The subjects consisted of 115 schizophrenia patients (70 males, 45 females) and 117 controls (54 males, 63 females). For genotype-altruism correlations, 209 Han Chinese students (96 males, 113 females, age 22.5±2.4) were recruited from Beijing Normal University. All subjects were unrelated, and genomic DNA was extracted from blood samples. For antipsychotics correlation studies, US Caucasian DNA and RNA samples from the Stanley Array Collection were extracted from postmortem dorsolateral prefrontal cortex (DLPFC) gray matter (Brodmann’s area 46) of 35 schizophrenics (26 males, 9 females) and 35 control subjects (26 males, 9 females). All samples were successfully extracted and used in further analysis.

To sequence the 3,551-bp segment flanked by SNPs rs6556547 and rs187269 in *GABRB2*, two genomic DNA regions were PCR-amplified to serve as first PCR templates for subsequent nested-PCR. The nested-PCR approach was adopted to increase amplification specificity. Nested-PCR and SNP genotyping by resequencing of the nested PCR products were carried out as described [15]. Primers for the PCR and sequencing reactions are given in Table S1. The genotyping completion rates were over 99% for the tested SNPs.

### Altruism Assessment

The self-report altruism scale [36] in the form of a questionnaire was used to assess altruistic attitudes, where altruistic behavior was identified by frequency of actions taken during the past year in 17 situations such as giving directions, helping people in need, and lending money on a scale of 1 (never) to 5 (very often).

### Statistical Analysis

Exact test of Hardy-Weinberg Equilibrium (HWE) was performed on SNPs in the control samples using GENEPOP 4.0 as described [6], and linkage disequilibrium (LD) was calculated using the DnaSP v5 program [37]. Pairwise haplotypes were inferred from the SNP genotypes of individual subjects using PHASE version 2.1 [38], and haplotypes with less than 1% frequencies were not included in the calculations. Genetic correlations with antipsychotics dosage, PANSS score, and altruistic tendency were analyzed using the linear-by-linear association test, Mann-Whitney U test and linear regression tests in the SPSS v11.5 package (SPSS, Chicago, IL, USA). Power estimates were performed using the PS program, version 3.0 [39]. Disease association was analyzed using the likelihood ratio statistic (LRS) test in UNPHASED program version 3.1.3 [40]. Permutation and resampling tests were chosen as validation tests. A global permutation test from UNPHASED employing 10,000 permutations for each SNP or haplotype with a *p*-value from the LRS test of less than 0.05 was performed. For resampling tests, 1,000 resampled datasets for each trait were generated by random extraction with replacement. Linear regression was performed for each SNP in each resampled dataset and the number of datasets per 1,000 sets showing significant correlation (*p*<0.05) were recorded. *GABRB2* isoforms expression and effects of sex on antipsychotics dosage, PANSS score, and altruism score were also analyzed using the Mann-Whitney U test; effects of age on the various traits were analyzed using Pearson’s correlation test.

## Results

The four SCZ-associated SNPs rs6556547 (S1), rs1816071 (S3), rs1816072 (S5) and rs187269 (S29) used previously in our analysis of positive selection [15], sequentially contained in a 3,551-bp fragment in *GABRB2*, were analyzed in the present study. The SNP code numbers in parentheses are adopted from our previous designations [12], [15]. No deviation from HWE (*p*>0.05) was detected for any of these SNPs in either the Chinese or US cohorts. Only the SNPs S3 and S5 in the US cohort but not in the Chinese cohort were completely linked, based on pairwise LD r^2^ = 0.94 for the US samples (Figure S1). Moreover, neither age nor sex exerted significant effects on PANSS score, antipsychotics dosage or altruism score.

### *GABRB2* Association with PANSS Scores

**Figure 1:**
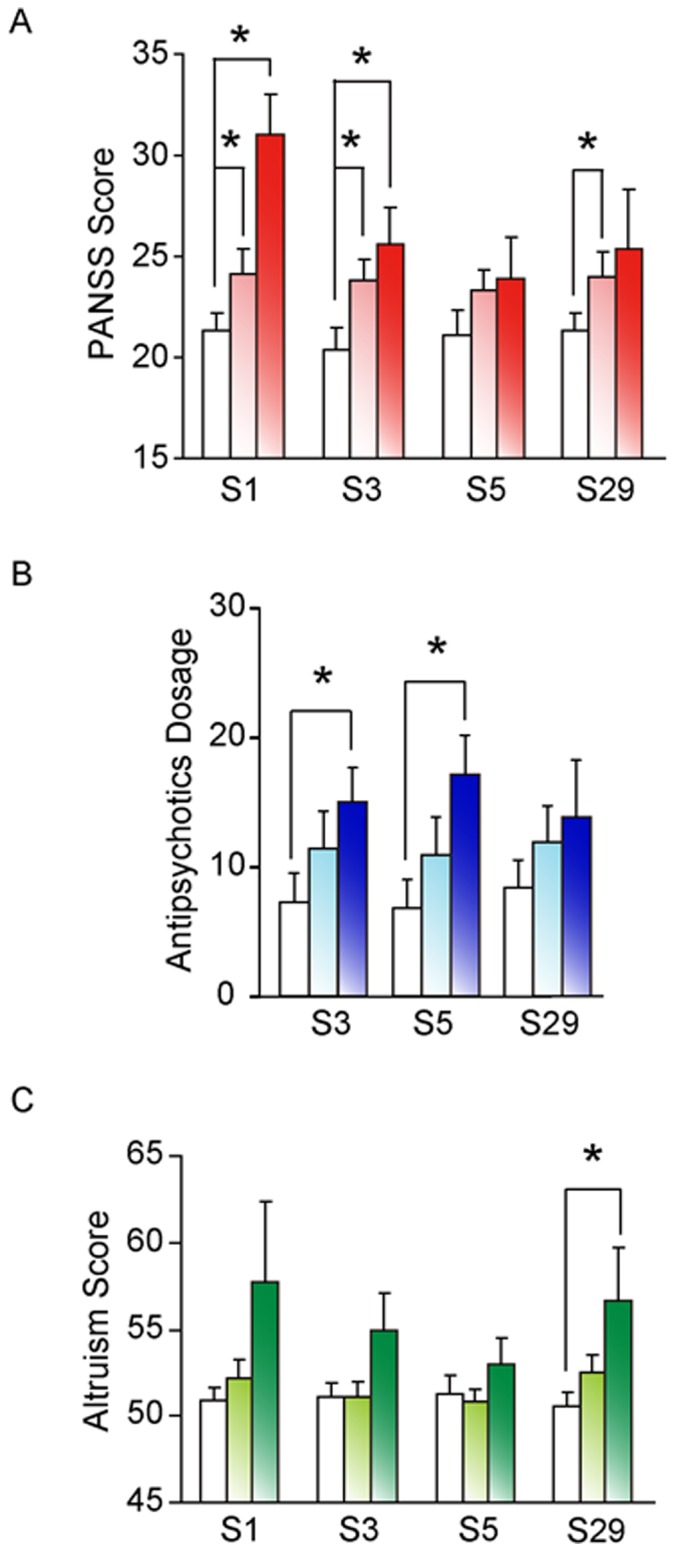
Post-hoc analyses of genetic effects on PANSS scores, antipsychotic dosage and altruism scores. PANSS positive scores (PANSS Score) of Chinese male schizophrenics (Part A), average antipsychotics dosage, in mg fluphenazine equivalents per day, of US schizophrenics (Part B) and altruism scores of healthy Chinese subjects (Parts C) are shown. The homozygous major genotype is shown by white column, heterozygous genotype by lighter colored column, and homozygous minor genotype by darker colored column, as mean ± standard error. Symbol *denotes significant difference (*p*<0.05 based on Mann-Whitney U test) between the two line-connected genotypes.

**Figure 2:**
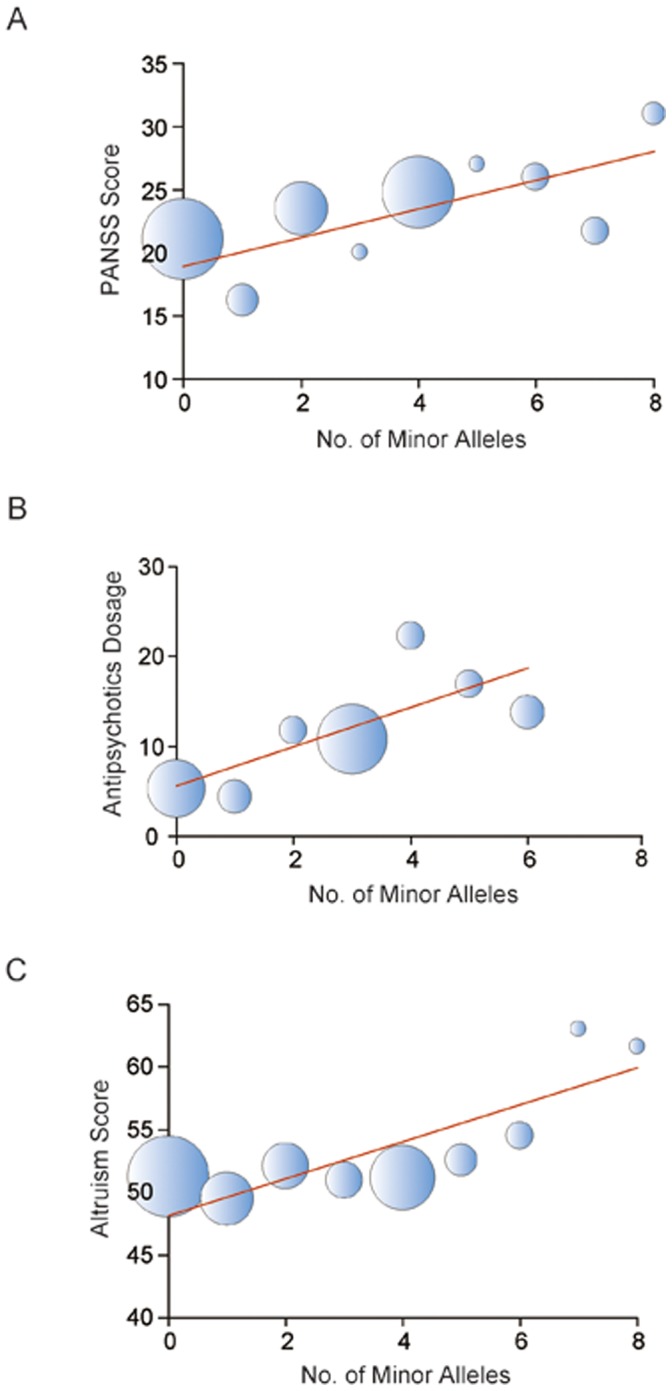
Quantitative correlation of minor allele load with PANSS scores, antipsychotics dosage and altruism scores. The bubble plot shows linear correlation between (A) PANSS positive score, (B) antipsychotics dosage and (C) altruism score and the number of minor alleles present at the four SNP locations (R^2^ = 0.51, 0.56 and 0.69, respectively, based on average score or dosage; *p* = 0.005, 0.022 and 0.033, respectively, based on linear regression analysis of original dataset). The bubbles represent average score or dosage, and their size represents the number of subjects.

Although the PANSS scores for positive symptoms (hereafter, unless stated otherwise, PANSS scores will refer to PANSS scores for positive symptoms) such as hallucinations and delusions for the full cohort of 70 male and 45 female Han Chinese SCZ patients were not significantly correlated with any *GABRB2* SNP genotype, the PANSS scores of the 70 male patients were significantly correlated with S1, S3 and S29 with *p* = 0.010, 0.010 and 0.041 respectively (Table 1), in each case with a positive correlation between the number of minor alleles and PANSS scores. The S29 correlation result did not pass the Bonferroni multiple test correction. In post-hoc analysis, higher PANSS scores were correlated with the S1 and S3 homozygous minor (*p* = 0.019 and 0.041, respectively) and the S1, S3 and S29 heterozygous (*p* = 0.043, 0.017 and 0.039, respectively; Figure 1A) genotypes compared to the corresponding homozygous major genotypes; and in the resampling tests, significant correlations (*p*<0.05) were observed between PANSS score and the S1, S3 and S29 genotypes for 737, 760 and 559 datasets, respectively, out of 1,000 resampled datasets. SNPs S1, S3 and S29 also showed significant correlation at the allele level (*p*<0.05), and four of the six inferred two-SNP haplotypes showed significant (*p*<0.05) correlations with PANSS score (Table S2). Moreover, the PANSS scores showed a propensity to increase linearly with increasing number of minor alleles present across the four SNP positions (Figure 2A; *p* = 0.005). Notably, for the full cohort as well as the two gender subgroups, none of the SNPs was significantly correlated (*p*>0.05) with the PANSS scores for negative symptoms such as emotional withdrawal or general symptoms such as anxiety, or with the total PANSS scores for all symptoms.

**Table 1:** Quantitative trait analyses of PANSS score, antipsychotics dosage and altruism score.

			PANSS				Antipsychotics dosage				Altruism			
			Male+Female		Male only		Male+Female		Male only		Male+Female		Male only	
			(n = 115)		(n = 70)		(n = 35)		(n = 26)		(n = 209)		(n = 96)	
SNP	M/m	Reference allele	Effectsize	*p*-value	Effectsize	*p*-value	Effectsize	*p*-value	Effectsize	*p*-value	Effectsize	*p*-value	Effectsize	*p*-value
S1	G/T	G	0.165	0.079	0.308	**0.010**	–	–	–	–	0.105	0.131	0.140	0.174
S3	A/G	A	0.130	0.166	0.312	**0.010**	0.273	0.111	0.260	0.194	0.089	0.209	0.111	0.281
S5	T/C	T	0.024	0.801	0.177	0.141	0.370	**0.031**	0.260	0.194	0.052	0.451	0.118	0.248
S29	T/C	T	0.133	0.156	0.246	**0.041**	0.190	0.268	0.136	0.497	0.159	**0.023**	0.170	0.099

Genetic effects on PANSS positive scores (PANSS Score) of Chinese schizophrenics; average antipsychotics dosage, in mg fluphenazine equivalents per day, of US schizophrenics; and altruism scores of healthy Chinese subjects are shown. The power estimates for the three cohorts were 0.6, 0.4 and 0.8, respectively. The effect sizes were represented by Pearson’s *r* values, and the *p*-values were obtained using the linear-by-linear association test in SPSS for additive effect testing. Significant *p* values (*p*<0.05) are shown in bold font.

**Figure 3:**
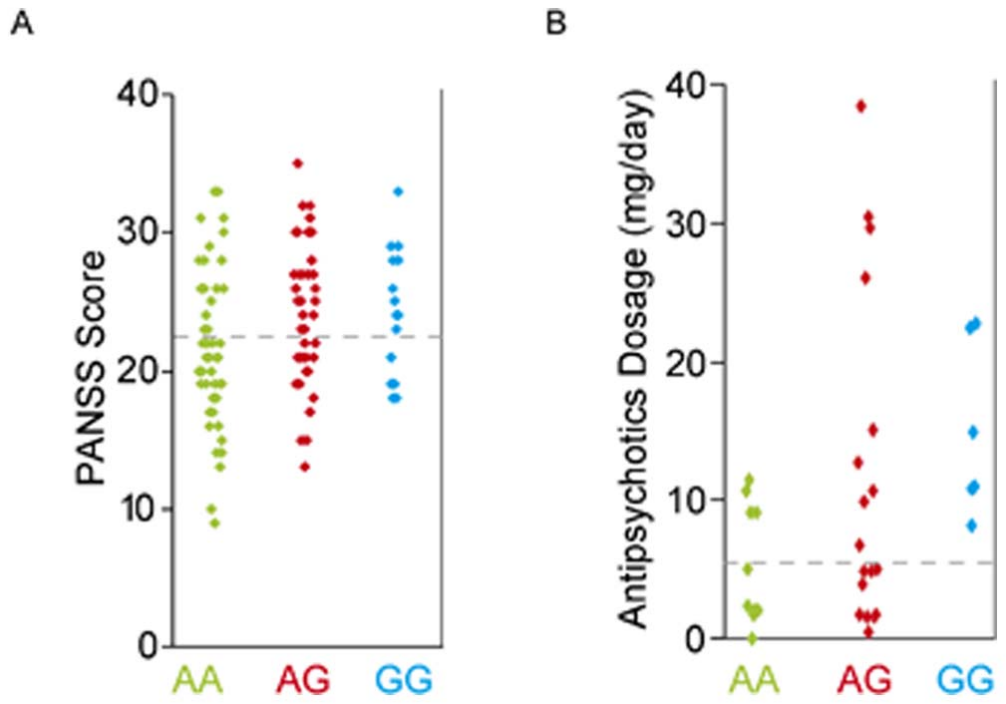
Two-tier grouping of PANSS scores and antipsychotics dosages. (A) PANSS positive scores for Chinese schizophrenics and (B) average daily antipsychotics dosages for US schizophrenics were sorted according to the genotypes AA, AG and GG of SNP S3. The dashed horizontal line in each instance divides the samples into two groups based on K-Means clustering for PANSS scores, or the clinically-recommended maintenance antipsychotics dosage of 5 mg/day.

**Figure 4:**
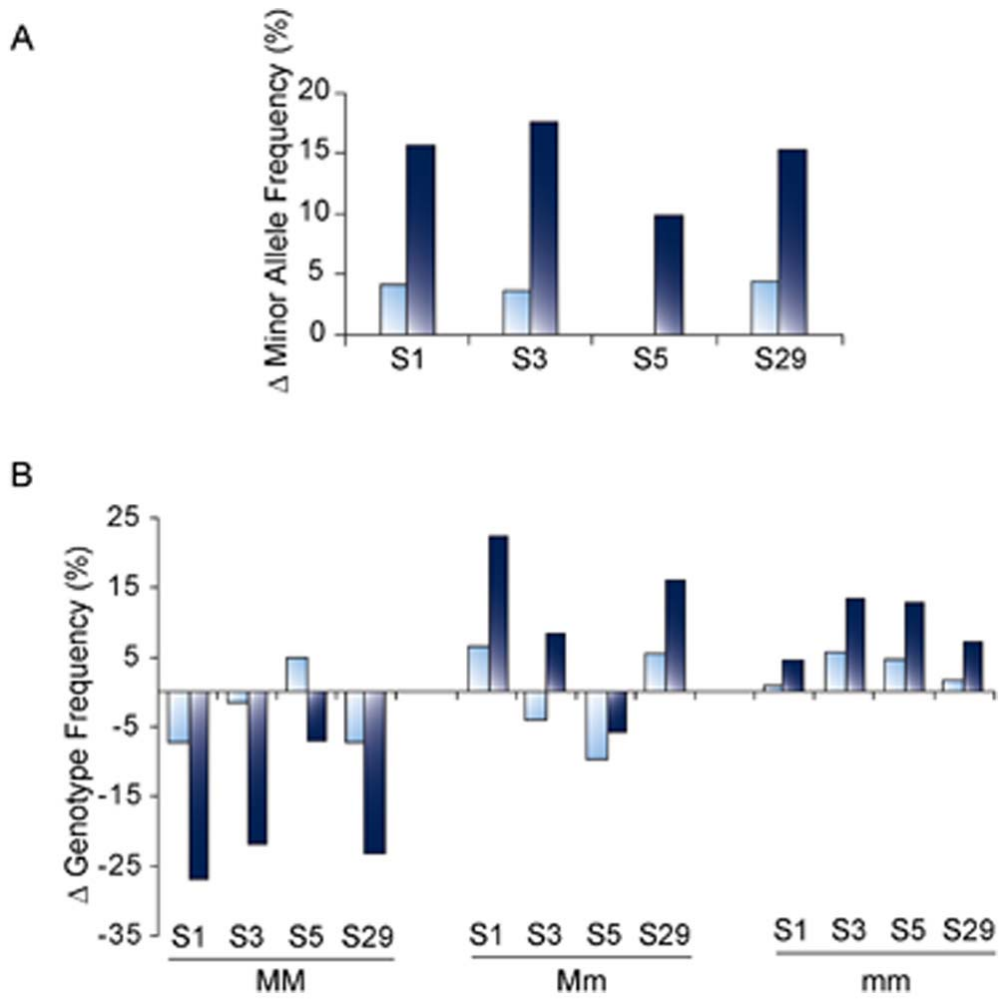
Relative increases in minor allele frequencies in PANSS score-based SCZ subgroups. Han Chinese SCZ patients were divided into ‘High’ and ‘Low’ PANSS positive score subgroups according to the K-Means clustering function. Case-control comparison was carried out between each patient subgroup and the healthy control group by means of the LRS test in UNPHASED. Part A: Excesses in minor allele frequencies in the case cohort over those in the control cohort are shown for SNPs S1, S3, S5 and S29. Part B: Excesses in homozygous major (MM), heterozygous (Mm) or homozygous minor (mm) genotype frequencies in the case cohort over those in the control cohort are shown for SNPs S1, S3, S5 and S29. In all instances, SCZ patients with ‘Low’ PANSS score are represented by light blue columns and SCZ patients with ‘High’ PANSS score by dark blue columns. All frequency data is given in Table S3 and the statistical data of association with SCZ is shown in Table 2.

The full cohort of 115 Han Chinese SCZ patients were separated into two groups based on their PANSS scores using the K-Means clustering function (Figure 3A). The full patient cohort, and the thus-separated ‘high PANSS score’ and ‘low PANSS score’ subgroups were each compared to the 117 healthy control subjects by means of the LRS test in UNPHASED. SNPs S1, S3 and S29 were significantly associated with SCZ in the full patient cohort in agreement with previous reports on association of *GABRB2* SNPs with SCZ in Han Chinese [5], [41]. Interestingly, many of the association *p*-values of these SNPs with SCZ in the high PANSS score subgroup were drastically decreased to less than 10% of the full cohort *p*-values; e.g., the full cohort *p*-values for SNP S1 of 0.005 and 0.014 for allele and genotype associations, respectively, were decreased to 0.0004 and 0.001 in the high PANSS score subgroup (Table 2). In contrast, there was no significant association of SCZ with any of the SNPs in the low PANSS score subgroup. The results reflected considerable increases in the frequencies of minor alleles and minor-allele-containing genotypes in the high PANSS score subgroup relative to control (Figure 4; Table S3). Such increases were negligible in the low PANSS score subgroup. Moreover, for all six pairwise haplotypes examined in Table 3, the *p*-values reflected highest significance in the high PANSS score subgroup, lower significance in the full cohort, and no significance (*p*>0.05) in the low PANSS score subgroup.

**Table 2:** *GABRB2* association in PANSS score-based SCZ subgroups.

	All (n = 115)				High score (n = 56)				Low score (n = 59)			
	Allele			Genotype	Allele			Genotype	Allele			Genotype
SNP	*p*	OR	95% CI	*p*	*p*	OR	95% CI	*p*	*p*	OR	95% CI	*p*
S1	**0.005**	2.04	1.24–3.38	**0.014**	**4×10^−4^**	2.82	1.59–4.99	**0.001**	0.288	1.41	0.75–2.65	0.548
S3	**0.014**	1.66	1.10–2.49	**0.042**	**0.001**	2.25	1.39–3.64	**0.006**	0.466	1.21	0.73–2.00	0.437
S5	0.293	1.22	0.84–1.78	0.177	0.080	1.51	0.95–2.38	0.114	0.988	1.00	0.63–1.58	0.430
S29	**0.010**	1.82	1.15–2.88	**0.044**	**0.001**	2.40	1.41–4.08	**0.008**	0.311	1.35	0.76–2.38	0.613

The Beijing SCZ cohort (‘All’) was partitioned into ‘High score’ and ‘Low score’ subgroups by applying the K-Means clustering function to the PANSS scores for positive symptoms. The *p*-values were obtained from case-control association analysis using the likelihood ratio test. Significant *p* values (*p*<0.05) that passed the global permutation test are shown in bold font. The odds ratio (OR) and its 95% confidence level (CI) were based on allele frequencies.

**Table 3:** *GABRB2* haplotype association in psychosis severity-based subgroups.

SNP composition^a^				PANSS score-based groups			Antipsychotics dosage-based groups		
S1	S3	S5	S29	All	High score	Low score	All	High dosage	Low dosage
X	X			**0.007**	**0.001**	0.417	–	–	–
X		X		**0.007**	**0.001**	0.320	–	–	–
X			X	**0.020**	**0.002**	0.569	–	–	–
	X	X		**0.008**	**0.001**	0.392	0.515	0.085	0.655
	X		X	**0.018**	**0.002**	0.514	0.256	0.111	0.393
		X	X	**0.007**	**0.003**	0.232	0.194	**0.034**	0.154

The PANSS score-based groups consist of the full Beijing SCZ cohort (‘All’) and its partitioned ‘High score’ and ‘Low score’ subgroups derived by applying the K-Means clustering function to the PANSS scores for positive symptoms. The antipsychotics dosage-based groups consist of the full US SCZ cohort (‘All’) and its partitioned ‘High dosage’ (>5 mg/day) and ‘Low dosage’ (≤5 mg/day) groups derived based on the daily standard maintenance antipsychotics dosage of 5 mg fluphenazine equivalent. The *p*-values were obtained from case-control association analysis using the likelihood ratio test. Significant *p* values (*p*<0.05) that passed the global permutation test are shown in bold font. ^a^Pairwise haplotypes with component SNPs indicated by X.

### *GABRB2* Association with Antipsychotics Dosage

The daily average antipsychotics dosages administered to US SCZ patients over their duration of illness were derived according to the equation:
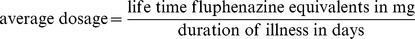



When SNPs S3, S5 and S29 were correlated with the daily average antipsychotics dosage (S1 was not included in the analysis on account of its low minor allele sample size), significant correlation was observed in the case of S5 with *p* = 0.031 (Table 1). The result did not pass the Bonferroni multiple test correction. Nevertheless, in post-hoc analysis, the S3 and S5 homozygous minor genotypes were associated with significantly higher average dosages than the homozygous major genotypes (*p* = 0.037 and 0.010, respectively; Figure 1B); and in the resampling test, 634 out of 1,000 resampled datasets were significant (*p*<0.05) for S5 genotype correlation with antipsychotics dosage. SNP S5 also showed significant correlation at the allele level (*p*<0.05), and the inferred pairwise haplotypes S3–S5 and S5–S29 were significantly correlated with antipsychotics dosage (Table S2). Moreover, the average dosage increased linearly with increasing number of minor alleles present across the three SNP positions (Figure 2B; *p* = 0.022).

**Figure 5:**
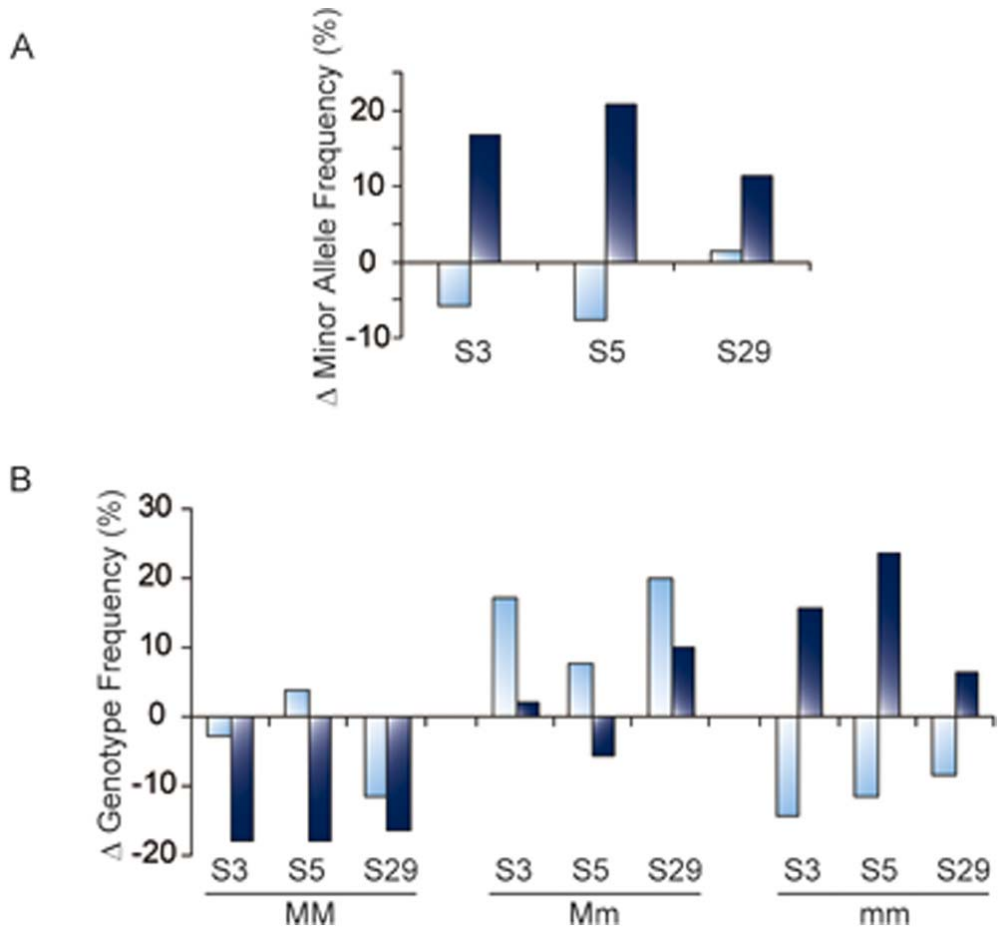
Relative increases in minor allele frequencies in antipsychotics dosage-based SCZ subgroups. US SCZ patients were divided into ‘High’ and ‘Low’ antipsychotics dosage subgroups based on the criterion of 5 mg/day fluphenazine equivalent. Case-control comparison was carried out between each patient subgroup and the healthy control group by means of the LRS test in UNPHASED. Part A: Excesses in minor allele frequencies in the case cohort over those in the control cohort are shown for SNPs S3, S5 and S29. Part B: Excesses in homozygous major (MM), heterozygous (Mm) or homozygous minor (mm) genotype frequencies in the case cohort over those in the control cohort are shown for SNPs S3, S5 and S29. In all instances, SCZ patients with ‘Low’ antipsychotics dosage are represented by light blue columns and SCZ patients with ‘High’ antipsychotics dosage by dark blue columns. All frequency data is given in Table S3 and the statistical data of association with SCZ is shown in Table 4.

Based on the clinical recommendation of 1–5 mg/day fluphenazine as a standard maintenance dose for SCZ and other psychotic disorders [42], patients could be separated into a ‘high-dosage’ >5 mg and a ‘low (or standard)-dosage’ ≤ 5 mg subgroups (Figure 3B). Of note, there were no subjects with S3, S5 or S29 homozygous minor genotype in the low dosage subgroup. Although there was no significant association of S3, S5 or S29 with SCZ for the full US cohort or low antipsychotics dosage subgroup, for the high dosage subgroup significant associations of S5 (Table 4; *p* = 0.034) as well as S5–S29 haplotype (Table 3; *p* = 0.034) with SCZ were observed, as well as an evident trend that the *p*-values of association were lower in the high dosage subgroup than in the low dosage subgroup. Moreover, the S3 and S5 minor allele and homozygous minor frequencies were considerably elevated in the high dosage subgroup over the controls (Figure 5; Table S3). These results were entirely in agreement with the analysis based on PANSS scores in the Chinese cohort (Tables 2 and 3).

**Table 4:** *GABRB2* association in antipsychotics dosage-based SCZ subgroups.

	All (n = 35)				High dosage (n = 20)				Low dosage (n = 15)			
	Allele			Genotype	Allele			Genotype	Allele			Genotype
SNP	*p*	OR	95% CI	*p*	*p*	OR	95% CI	*p*	*p*	OR	95% CI	*p*
S3	0.387	1.35	0.68–2.67	0.612	0.087	1.99	0.90–4.38	0.260	0.578	0.77	0.31–1.94	0.122
S5	0.297	1.44	0.73–2.85	0.559	**0.034**	2.34	1.06–5.19	0.098	0.450	0.70	0.27–1.80	0.222
S29	0.365	1.39	0.68–2.83	0.456	0.222	1.67	0.74–3.78	0.464	0.886	1.07	0.42–2.74	0.192

Based on the average daily antipsychotics dosage of 5 mg fluphenazine equivalent, the US SCZ cohort (‘All’) was divided into ‘High dosage’ (>5 mg/day) and ‘Low dosage’ (≤5 mg/day) groups. The *p*-values were obtained from case-control association analysis using the likelihood ratio test. Significant *p* values (*p*<0.05) that passed the global permutation test are shown in bold font. The odds ratio (OR) and its 95% confidence level (CI) were based on allele frequencies.

**Figure 6:**
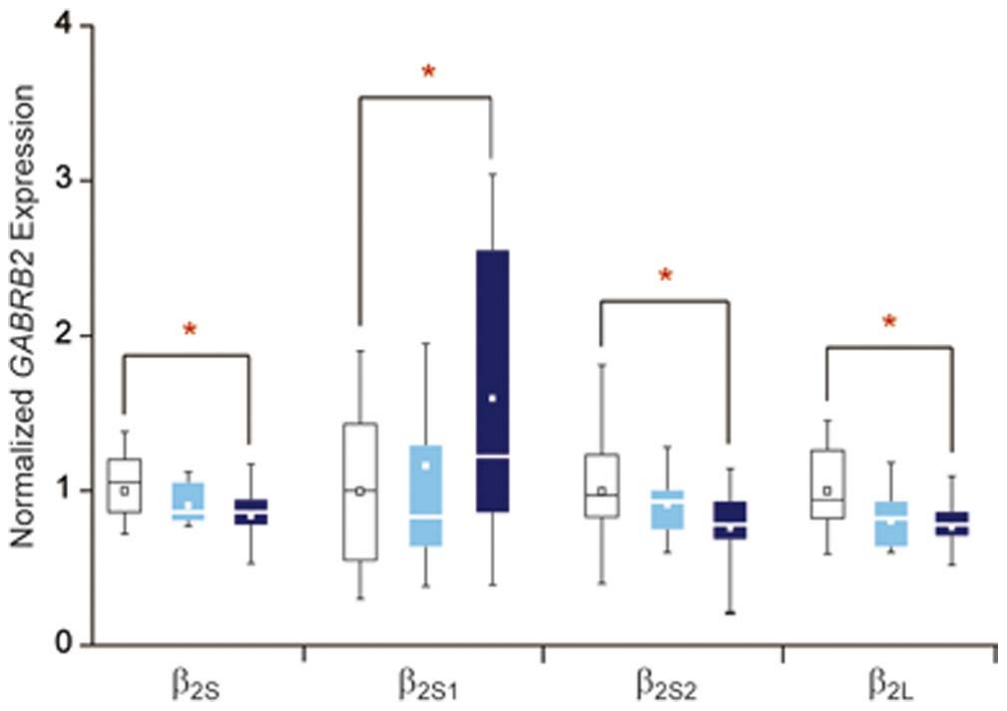
Differential expression of *GABRB2* isoforms in antipsychotics dosage-based SCZ subgroups. US SCZ patients were divided into a ‘High’ (>5 mg/day) antipsychotics dosage subgroup (n = 20) and a ‘Low’ (≤5 mg/day) subgroup (n = 15). The expression levels of β_2S_, β_2S1_, β_2S2_ and β_2L_ in the healthy control group (white), subgroup of patients with ‘Low’ average antipsychotics dosage (light blue), or subgroup of patients with ‘High’ average antipsychotics dosage (dark blue) are shown in box-and-whisker representation with a specified percentile range of 5%–95%. For each isoform, the mean expression level in the healthy control group was employed to normalize the data for that isoform. The small square within each box indicates the mean expression level for that data group. Symbol * denotes significant difference in expression between the two line-connected groups.

Previously, the US samples showed correlations between SCZ and expression of *GABRB2* isoforms, including the two long recognized short β_2S_ and long β_2L_ isoforms [10] as well as the two novel isoforms β_2S1_ and β_2S2_ [11]. Figure 6 shows that, upon partition of the US SCZ samples into the high and low antipsychotics dosage subgroups, significant differences in *GABRB2* expression with respect to all four isoforms were observed between healthy controls and the high dosage subgroup, but not between controls and the low dosage subgroup.

### *GABRB2* Association with Altruism

When the altruistic behavior of normal subjects was assessed based on the self-report altruism scale [36], significant association was observed between altruistic behavior and S29 (*p* = 0.023), based on an additive effect test with a positive correlation between the number of minor alleles and altruism scores (Table 1). The result did not pass the Bonferroni multiple test correction. Nevertheless, in post-hoc analysis, subjects with the homozygous minor genotype of S29 were significantly more altruistic than subjects with the homozygous major genotype (*p* = 0.043; Figure 1C), and in the resampling test, 619 out of 1,000 resampled datasets showed significant correlation (*p*<0.05) between S29 genotype and altruism score. SNP S29 also showed significant correlation with altruism score at the allele level (*p* = 0023; Table S2). Moreover, the altruism scores showed a propensity to increase linearly with increasing number of minor alleles present across the four SNP positions (Figure 2C; *p* = 0.033).

## Discussion

The segment flanked by SNPs rs6556547 (S1) and rs187269 (S29) in *GABRB2* was earlier shown by us to play important roles in the etiology of the neuropsychiatric disorders SCZ and BPD [5], [6], [10], [11]. The positive selection of a number of SNPs in this segment suggests *GABRB2* involvement in not only injurious mental disease processes, but also the development of traits and capabilities that benefit human fitness during emergence of the human lineage. This would be in line with an important role of the *GABRB2* gene in cognitive functions that both underlie SCZ etiology and are integral to human evolution. The present study was accordingly directed to a parallel examination of altruism and schizophrenic psychosis.

### Association of *GABRB2* with Psychosis in SCZ

PANSS is a useful measure of psychosis severity in SCZ [34], and PANSS scores reflecting severity of positive symptoms such as hallucinations and delusions were found to be significantly associated with SNPs S1, S3 and S29 in the male Han Chinese SCZ cohort (*p* = 0.010, 0.010 and 0.041, respectively; Table 1). These findings were corroborated to some degree by the association of SNP S5 with average daily antipsychotics dosage given to SCZ patients in a US cohort (*p* = 0.031; Table 1). The average daily antipsychotics dosage reflects relative effective dose within the cohort, and even though effective dosage can be influenced by other factors including prescribing practice of psychiatrists and differences in inter-individual drug response, it is in general indicative of psychosis severity. Therefore, these first reports of association between *GABRB2* and psychosis in SCZ were cross-corroborated using two different measures of psychosis in two different ethnic populations. However, it should be noted that the S29 association with PANSS score and S5 association with antipsychotics dosage did not pass Bonferroni correction, although this is mitigated by the test’s tendency for over-correction of potentially significant results and the presence of supporting evidence for correlation in the post-hoc results. In addition, the small sample sizes used reduce the statistical power and so the actual significance of the findings requires confirmation using increased sample sizes.

The two-tiered partitioning based on PANSS scores and antipsychotics dosages allowed a dimorphic subgrouping of SCZ patients according to psychotic severities. The results in Tables 2, 3, 4 showed that the strength of *GABRB2* association significantly segregated with the patient subgroups having the more severe psychosis symptoms (for example, *p* = 0.001–0.003 vs. *p* = 0.232–0.569 for pairwise haplotypes in the patient subgroup with high PANSS scores vs. subgroup with low PANSS scores), and the association was due to increased frequencies of minor alleles in these patient subgroups relative to control (Figures 4 and 5). Importantly, this finding suggested that the association of *GABRB2* with SCZ is centered at the core symptom of psychosis of the disorder. Similarly, in Figure 6, the expression levels of all four *GABRB2* isoforms in the high antipsychotics dosage SCZ patient subgroup, but not in the low dosage subgroup, significantly departed from the controls (*p* = 0.023, 0.047, 0.011 and 0.004 for the β_2S_, β_2S1_, β_2S2_ and β_2L_ isoforms, respectively), in accord with the effects of *GABRB2* being associated with the psychosis endophenotype. This association between *GABRB2* with psychosis severity in SCZ is corroborated by parallel observations on other SCZ-candidate genes including: effects of *COMT* [20] and *DISC* [43] on psychosis severity in SCZ; *GABRB2* [26] and *COMT* [44] on occurrence of psychosis in BPD; *COMT*, *DAOA* and *HTR2A* on occurrence of psychosis in Alzheimer’s disease [22], [23], [45]; and dopamine dysfunction on occurrence of psychosis in SCZ [46]. Such multi-disease associations with the same psychosis endophenotype strongly support the possibility that the involvements of these genes in psychiatric disorders are mediated at least in part by their contribution to psychosis. In aside, the present findings implies that sample homogeneity could be increased by considering psychosis severity, and this can in turn increase the statistical power of future genetic association studies on SCZ.

### Genetic Overlap between the Social Cognition-Related Phenotypes of Psychosis and Altruism

Altruism refers to behavior that confers a benefit to others at a cost to oneself, motivated less by self-interest than notions of fairness and social obligation. Both altruism and psychosis display significant genetic roots: the heritability of altruism is as high as 0.50 [32] and the heritabilities of the psychotic diseases SCZ and BPD are well known [47]. Moreover, both altruism and psychosis are intrinsically linked to social cognition, and a psychosis to altruism continuum has in fact been employed to furnish a ‘psychoticism’ measure of individual personality [48]. Therefore, although hitherto altruism has never been associated explicitly with either psychiatric disorders or GABAergic function, it cannot be ruled out that altruism and clinical psychotic behavior share common genetic elements. In this regard, the SNPs in *GABRB2* that are strongly associated with SCZ provide uniquely advantageous genetic probes to search for common genetic elements between altruism and psychosis. The present study accordingly examined the possible relationships between altruism and these SCZ-associated SNPs. The results showed that the minor allele of S29 relative to the major allele (*p* = 0.023; Table 1, Figure 1C), was associated significantly with altruistic behavior in healthy Han Chinese. Although the result did not pass the Bonferroni correction, the correlation is nevertheless supported by the post-hoc results. Therefore, *GABRB2* plays a significant role not only in SCZ but also in the genetic predisposition of individuals to altruism. In particular, the combined minor allele load of the tested SNPs was predictive for both psychosis (*p* = 0.005; Figure 2A) and altruism (*p* = 0.033; Figure 2C), highlighting the genetic overlap as well as the collective importance of these minor alleles in the two phenotypes. Notably, the Val158Met polymorphism of the SCZ-associated *COMT* gene was also found to be associated with both psychotic symptoms [20], [44], [45] and altruism [49], with the major Val allele favoring both increased risk of psychosis and enhanced inclination for altruism.

Insofar that the social cognitive functions in psychosis and altruism may be implemented by neuronal gene products of *cognition* genes, the possibility arises that *GABRB2* of the GABAergic system and *COMT* of the dopaminergic system came to reveal themselves as SCZ-susceptibility genes as well as altruism genes on account of their putative roles as cognition genes. This would explain the striking involvement of the same polymorphisms of *GABRB2* and of *COMT* in both SCZ-psychosis and altruism, which may be viewed respectively as psychiatric and psychological manifestations of social cognition.

In conclusion, the present study demonstrated that *GABRB2* is significantly associated with altruism and psychosis in SCZ. Since the *GABRB2*-SCZ associations were strong among schizophrenics with severe psychosis but not schizophrenics with only mild psychosis based on the PANSS scores, the findings indicated that the association of *GABRB2* with SCZ is centered at the core symptom of psychosis of the disorder. Furthermore, the associations of *GABRB2* with both psychosis and altruism suggest its fundamental association with the entire psychiatric to psychological spectrum of social cognition, thus establishing *GABRB2* involvement at the level of social cognition. Given the power of advances in social cognition in driving the evolution of the human lineage from its primate roots, *GABRB2* as a key genetic element of social cognition would provide an ample evolutionary rationale for the strong positive selection observed to be acting on this gene.

## Supporting Information

Figure S1 **Pairwise linkage disequilibrium plots for US and Beijing control groups.** LD between all possible pairs of SNPs are measured by *D’* (shown by numbers in each square) and *r^2^* (range indicated by grey-scale shading).
(TIF)

Table S1 Primers for PCR and sequencing of the 3.55 kb *GABRB2* fragment.
(DOC)

Table S2 *GABRB2* correlations with PANSS score, antipsychotics dosage and altruism score.
(DOC)

Table S3 Frequency data of *GABRB2* SNPs in (A) Beijing Chinese and (B) US Caucasian cohorts.
(DOC)
