# Supplementary figures and images for: Social Cognitive Role of Schizophrenia Candidate Gene GABRB2
Source: PLoS One. 2013 Apr 24;8(4):e62322. doi: 10.1371/journal.pone.0062322 (PMC3634734; doi:10.1371/journal.pone.0062322)

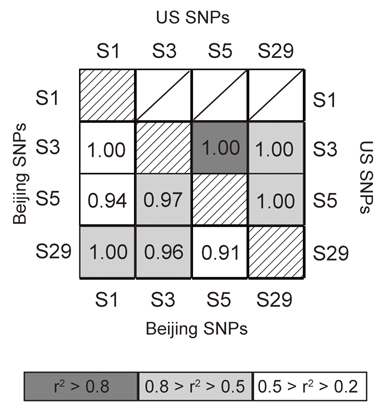

Supplement: Figure S1 — Pairwise linkage disequilibrium plots for US and Beijing control groups. LD between all possible pairs of SNPs are measured by D’ (shown by numbers in each square) and r2 (range indicated by grey-scale shading). (TIF) [file pone.0062322.s001.tif]
