# Supplementary material for: Social Cognitive Role of Schizophrenia Candidate Gene GABRB2
Source: PLoS One. 2013 Apr 24;8(4):e62322. doi: 10.1371/journal.pone.0062322 (PMC3634734; doi:10.1371/journal.pone.0062322)
Supplement: Table S1 — Primers for PCR and sequencing of the 3.55 kb GABRB2 fragment. (DOC) [file pone.0062322.s002.doc]

**Table S1.** Primers for PCR and sequencing of the 3.55 kb *GABRB2* fragment

| DNA fragment | Orientation | Sequence (5’ – 3’) |
| --- | --- | --- |
| First PCR |  |  |
| Fragment A | Forwards | ATGGAGGAAAGGTCCATATCTAGT |
|  | Reverse | GGTCATTGTGAGGACAGTTG |
| Fragment B | Forwards | AGGCTGCCAGTGCCAACAAT |
|  | Reverse | AGACAATGCCTAATGTCCTCTGG |
| Nested PCR |  |  |
| Fragment A | Forwards | GGGAATGGTGCTCAGTAAAC |
|  | Reverse | CTTAATAGCTGGAAAGGTGAT |
| Fragment B | Forwards | AGCACTTGCTGCACTAA |
|  | Reverse | GTTTCCAAGGGCCATACTTAAAAT |
| Sequencing |  |  |
| Fragment A | - | TTCTCGTGTCCTCTTTATAGC |
|  | - | CCTCTAAGCTGTAATCGGAAGGTA |
| Fragment B | - | AGCACTTGCTGCACTAA |
