# Supplementary material for: Social Cognitive Role of Schizophrenia Candidate Gene GABRB2
Source: PLoS One. 2013 Apr 24;8(4):e62322. doi: 10.1371/journal.pone.0062322 (PMC3634734; doi:10.1371/journal.pone.0062322)
Supplement: Table S2 — GABRB2 correlations with PANSS score, antipsychotics dosage and altruism score. (DOC) [file pone.0062322.s003.doc]

**Table S2.** *GABRB2* correlations with PANSS score, antipsychotics dosage and altruism score.

| SNP composition^a^ | | | | *p*-values | | |
| --- | --- | --- | --- | --- | --- | --- |
| S1 | S3 | S5 | S29 | PANSS | Antipsychotics | Altruism |
| X |  |  |  | **0.010** | - | 0.130 |
|  | X |  |  | **0.008** | 0.069 | 0.281 |
|  |  | X |  | 0.097 | **0.015** | 0.531 |
|  |  |  | X | **0.029** | 0.440 | **0.023** |
| X | X |  |  | **0.019** | - | 0.317 |
| X |  | X |  | **0.035** | - | 0.310 |
| X |  |  | X | **0.035** | - | 0.077 |
|  | X | X |  | **0.010** | **0.047** | 0.476 |
|  | X |  | X | 0.069 | 0.257 | 0.069 |
|  |  | X | X | 0.089 | **0.034** | 0.084 |

The *p*-values were obtained by correlating single SNPs or inferred pairwise haplotypes with PANSS positive score of male Chinese schizophrenics, antipsychotics dosage of US Caucasian schizophrenics, and altruism score of healthy Chinese subjects using the Mann Whitney U test or Kruskal-Wallis H test. Significant *p* values (*p* < 0.05) are shown in bold font, and marginally significant values (*p* < 0.10) are underlined.

^a^ Single SNPs or pairwise haplotypes with component SNPs indicated by X.
