# Supplementary material for: Social Cognitive Role of Schizophrenia Candidate Gene GABRB2
Source: PLoS One. 2013 Apr 24;8(4):e62322. doi: 10.1371/journal.pone.0062322 (PMC3634734; doi:10.1371/journal.pone.0062322)
Supplement: Table S3 — Frequency data of GABRB2 SNPs in (A) Beijing Chinese and (B) US Caucasian cohorts. (DOC) [file pone.0062322.s004.doc]

**Table S3.** Frequency data of *GABRB2* SNPs

A. Beijing Chinese cohorts

|  | Frequency (%) | | | | | | | | | | | |
| --- | --- | --- | --- | --- | --- | --- | --- | --- | --- | --- | --- | --- |
|  | m | | | | M/M | | | | m/m | | | |
|  | CON | SCZ | | | CON | SCZ | | | CON | SCZ | | |
|  |  | All | Low score | High score |  | All | Low score | High score |  | All | Low score | High score |
| SNP | (n = 117) | (n =115) | (n = 59) | (n =56) |  |  |  |  |  |  |  |  |
| S1 | 11.94 | 21.74 | 16.1 | 27.68 | 76.92 | 60.00 | 69.79 | 50.00 | 0.85 | 3.48 | 1.69 | 5.36 |
| S3 | 24.34 | 34.78 | 27.97 | 41.96 | 57.52 | 46.09 | 55.93 | 35.71 | 6.19 | 15.65 | 11.86 | 19.64 |
| S5 | 36.52 | 41.30 | 36.44 | 46.43 | 39.13 | 38.26 | 44.07 | 32.14 | 12.17 | 20.87 | 16.95 | 25.00 |
| S29 | 15.95 | 25.65 | 20.34 | 31.25 | 71.55 | 56.52 | 64.41 | 48.21 | 3.45 | 7.83 | 5.08 | 10.71 |

B. US Caucasian cohorts

|  | Frequency (%) | | | | | | | | | | | |
| --- | --- | --- | --- | --- | --- | --- | --- | --- | --- | --- | --- | --- |
|  | m | | | | M/M | | | | m/m | | | |
|  | CON | SCZ | | | CON | SCZ | | | CON | SCZ | | |
|  |  | All | Low dosage | High dosage |  | All | Low dosage | High dosage |  | All | Low dosage | High dosage |
| SNP | (n = 35) | (n = 35) | (n = 15) | (n =20) |  |  |  |  |  |  |  |  |
| S3 | 35.71 | 42.86 | 30.00 | 52.50 | 42.86 | 31.43 | 60.00 | 25.00 | 14.29 | 17.14 | 0.00 | 30.00 |
| S5 | 34.29 | 42.86 | 26.67 | 55.00 | 42.86 | 34.29 | 53.33 | 25.00 | 11.43 | 20.00 | 0.00 | 35.00 |
| S29 | 28.57 | 35.71 | 30.00 | 40.00 | 51.43 | 37.14 | 60.00 | 35.00 | 8.57 | 8.57 | 0.00 | 15.00 |

(A) The Beijing SCZ cohort (‘All’) was partitioned into ‘High score’ and ‘Low score’ subgroups by applying the K-Means clustering function to the PANSS scores for positive symptoms. (B) Based on the average daily antipsychotics dosage of 5 mg fluphenazine equivalent, the US SCZ cohort (‘All’) was divided into ‘High dosage’ (> 5mg/day) and ‘Low dosage’ (≤ 5mg/day) groups.

Abbreviations: m, minor allele; M/M, homozygous major; m/m, homozygous minor; CON, control cohort; SCZ, schizophrenia cohort
